# Supplementary material for: Colonization with extended-spectrum β-lactamase and carbapenemase-producing Enterobacterales in Ethiopia: A systematic review and meta-analysis
Source: PLoS One. 2025 Apr 1;20(4):e0316492. doi: 10.1371/journal.pone.0316492 (PMC11960885; doi:10.1371/journal.pone.0316492)
Supplement: S2 Table — (DOCX) [file pone.0316492.s002.docx]

| 1 | (Gastrointestinal infections OR fecal carriage OR colonization OR asymptomatic infections).ti. |
| --- | --- |
| 2 | (Antimicrobial resistance OR Antibiotic resistance OR Betalactam resistance OR Antibiotic susceptibility OR Antibiotic sensitivity OR Beta lactama* OR carbapenema* OR multi-drug resistance carbapenem resistance OR Extended-spectrum β-lactamase).ab. |
| 3 | (Gram-negative bacilli, bacterial).sh. |
| 4 | (Enterobacterales OR Enterobacteriaceae OR Escherichia coli, OR Klebsiella pneumonia).sh. |
| 5 | (Human* OR child* neona*OR adult* OR patient*).tw. |
| 6 | Ethiopia.sh. |
| 7 | 3 OR 4 |
| 8 | 1 AND 2 |
| 9 | 5 AND 6 AND 7 AND 8 |
| 10 | ((((("gastrointestinal"[All Fields] OR "gastrointestinally"[All Fields] OR "gastrointestine"[All Fields]) AND "infections"[MeSH Terms]) OR "asymptomatic infections"[MeSH Terms]) AND ("drug resistance, microbial"[MeSH Terms] OR "drug resistance, microbial"[MeSH Terms] OR (("anti-bacterial agents"[Pharmacological Action] OR "anti-bacterial agents"[MeSH Terms] OR ("anti-bacterial"[All Fields] AND "agents"[All Fields]) OR "anti-bacterial agents"[All Fields] OR "antibiotic"[All Fields] OR "antibiotics"[All Fields] OR "antibiotic s"[All Fields] OR "antibiotical"[All Fields]) AND "disease susceptibility"[MeSH Terms]) OR "beta lactama*"[MeSH Terms] OR "drug resistance, multiple"[MeSH Terms] OR ("Extended-spectrum"[All Fields] AND "beta lactamases"[MeSH Terms])) AND ("Gram-negative"[All Fields] AND ("bacillis"[All Fields] OR "bacillus"[MeSH Terms] OR "bacillus"[All Fields] OR "bacilli"[All Fields]))) OR ("enterobacteriaceae"[MeSH Terms] OR "escherichia coli"[MeSH Terms] OR (("klebsiella"[MeSH Terms] OR "klebsiella"[All Fields] OR "klebsiellae"[All Fields] OR "klebsiellas"[All Fields]) AND "pneumonia"[MeSH Terms]))) AND ("human*"[MeSH Terms] OR "child*"[MeSH Terms] OR "neona*"[MeSH Terms] OR "adult*"[MeSH Terms] OR "patient*"[MeSH Terms]) AND "ethiopia"[MeSH Terms] |

**Table, Supplementary file 2, example of searching strategy from PubMed**
