# Supplementary material for: Colonization with extended-spectrum β-lactamase and carbapenemase-producing Enterobacterales in Ethiopia: A systematic review and meta-analysis
Source: PLoS One. 2025 Apr 1;20(4):e0316492. doi: 10.1371/journal.pone.0316492 (PMC11960885; doi:10.1371/journal.pone.0316492)
Supplement: S3 Table — (DOCX) [file pone.0316492.s003.docx]

Supplementary file 3 Table, for quality assessment of the studies included in the systematic review and meta-analysis

| Author, Year | Q1 | Q2 | Q3 | Q4 | Q5 | Q6 | Q7 | Q8 | Q9 | Score out of 9 | | Remark | Date of extraction | Authors who extracted |
| --- | --- | --- | --- | --- | --- | --- | --- | --- | --- | --- | --- | --- | --- | --- |
| Temsegen et al, 2023 [1] | Yes | No | No | Yes | Yes | Yes | Yes | Yes | UN | 6 | | Included | May 06/2024 | MT |
| Tola et al, 2021 [2] | Yes | No | No | Yes | Yes | Yes | Yes | Yes | Yes | 7 | | Included | May 06/2024 | MT |
| Zakir et al, 2021 [3] | Yes | Yes | No | Yes | Yes | Yes | Yes | Yes | Yes | 8 | | Included | May 06/2024 | MT |
| Zenebe et al, 2023 [4] | Yes | Yes | Yes | Yes | Yes | Yes | Yes | Yes | No | 8 | | Included | May 06/2024 | MT |
| Shenkute et al, 2022 [5] | Yes | No | Yes | Yes | Yes | Yes | Yes | Yes | Yes | 8 | | Included | May 06/2024 | MT |
| Kiros et al, 2023 [6] | Yes | No | Yes | Yes | Yes | Yes | Yes | Yes | UN | 7 | | Included | May 07/2024 | MT |
| Amare et al, 2022 [7] | UN | Yes | No | Yes | Yes | Yes | Yes | Yes | No | 6 | | Included | May 07/2024 | MT |
| Diriba et al, 2020 [8] | Yes | Yes | No | Yes | Yes | Yes | Yes | UN | Yes | 7 | | Included | May 07/2024 | MA |
| Aklilu et al, 2022 [9] | No | Yes | Yes | Yes | Yes | No | Yes | Yes | UN | 6 | | Included | May 06/2024 | MA |
| Bayleyegn et al, 2020 [10] | Yes | No | No | Yes | Yes | Yes | Yes | Yes | Yes | 7 | | Included | May 06/2024 | MA |
| Worku et al, 2022 [11] | No | No | Yes | Yes | Yes | Yes | Yes | Yes | Yes | 7 | | Included | May 06/2024 | MA |
| Desta et al, 2016 [12] | Yes | No | No | Yes | Yes | Yes | Yes | Yes | No | 6 | | Included | May 06/2024 | MA |
| Wolde et al, 2024 [13] | Yes | No | Yes | Yes | Yes | Yes | Yes | Yes | No | 7 | | Included | May 07/2024 | GG |
| Mekonnen et al, 2023 [14] | Yes | Yes | Yes | Yes | Yes | Yes | Yes | Yes | UN | 8 | | Included | May 07/2024 | GG |
|  | Q1 | Q2 | Q3 | Q4 | Q5 | Q6 | Q7 | Q8 | Q9 | Q10 | Q11 | Score out of 11 |  |  |
| Amsalu et al, 2024 [15] | No | Yes | Yes | Yes | Yes | Yes | Yes | Yes | No | No | Yes | 8 & Included | May 08/2024 | MT & GG |

Note: UN, unclear; Q, question. The overall score is calculated by counting the number of Yes’s in each row.

Q1: Was the sample frame appropriate to address the target population?, Q2: Were study participants sampled in an appropriate way?, Q3: Was the sample size adequate?, Q4: Were the study subjects and the setting described in detail? Q5: Was the data analysis conducted with sufficient coverage of the identified sample?, Q6 : Were valid methods used for the identification of the condition?, Q7: Was the condition measured in a standard reliable way for all participants? Q8: Was there appropriate statistical analysis?, and Q9: Was the response rate adequate, and if not, was the low response rate managed appropriately were for cross-sectional studies. However, the following questions were for cohort studies. Q1: Were the two groups similar and recruited from the same population?, Q2: Were the exposures measured similarly to assign people to both exposed and unexposed groups?, Q3: Was the exposure measured in a valid and reliable way?, Q4: Were confounding factors identified?, Q5: Were strategies to deal with confounding factors stated?, Q6: Were the groups/participants free of the outcome at the start of the study (or at the moment of exposure)?, Q7: Were the outcomes measured in a valid and reliable way? Q8: Was the follow up time reported and sufficient to be long enough for outcomes to occur?, Q9: Was follow up complete, and if not, were the reasons to loss to follow up described and explored?, Q10: Were strategies to address incomplete follow up utilized?, Q11:Was appropriate statistical analysis used?

**The number of studies we identified during the literature search and excluded during screening, along with the reason(s) for their exclusion.**

| Reasons for exclusion | Excluded studies | Stage of screening |
| --- | --- | --- |
| Not Ethiopian studies (n=43) | [16-58] | During title and abstract screening |
| Not similar topics (n=36) | [59-69],[70-85], [86-94] | During title and abstract screening |
| Reviews (systematic review and meta-analysis (n=14) | [95-108] | During title and abstract screening |
| Reported other than stool specimens (n=24) | [109-132] | During full text reviewing |
| Lack of full text or outcome (ESBL-PE or CPE) not reported (n=3) | [133-135] | During full text assessing for eligibility |

1. Temsegen W, Gorems K, Mekonnen M, Fufa D, Kassa T. Fecal Carriage of Extended-Spectrum β-Lactamase and Carbapenemase-Producing Enterobacteriaceae Among Oncology and Non-Oncology Patients at Jimma Medical Center in Ethiopia: A Comparative Cross-Sectional Study. *Cancer management and research*. 2023;**15**:1217-31.10.2147/cmar.S422376.

2. Tola MA, Abera NA, Gebeyehu YM, Dinku SF, Tullu KD. High prevalence of extended-spectrum beta-lactamase-producing Escherichia coli and Klebsiella pneumoniae fecal carriage among children under five years in Addis Ababa, Ethiopia. *PloS one*. 2021;**16**(10):e0258117.10.1371/journal.pone.0258117.

3. Zakir A, Regasa Dadi B, Aklilu A, Oumer Y. Investigation of Extended-Spectrum β-Lactamase and Carbapenemase Producing Gram-Negative Bacilli in Rectal Swabs Collected from Neonates and Their Associated Factors in Neonatal Intensive Care Units of Southern Ethiopia. *Infection and drug resistance*. 2021;**14**:3907-17.10.2147/idr.S333603.

4. Zenebe T, Eguale T, Desalegn Z, Beshah D, Gebre-Selassie S, Mihret A, et al. Distribution of ß-Lactamase Genes Among Multidrug-Resistant and Extended-Spectrum ß-Lactamase-Producing Diarrheagenic Escherichia coli from Under-Five Children in Ethiopia. *Infection and drug resistance*. 2023;**16**:7041-54.10.2147/idr.S432743.

5. Shenkute D, Legese MH, Yitayew B, Mitiku A, Engidaye G, Gebremichael S, et al. High Magnitude of Fecal Carriage of Extended-Spectrum Beta-Lactamase-Producing Enterobacteriaceae at Debre Berhan Comprehensive Specialized Hospital, Ethiopia. *Infection and drug resistance*. 2022;**15**:2445-58.10.2147/idr.S356807.

6. Kiros T, Belete D, Andualem T, Workineh L, Tilahun M, Eyayu T, et al. Carriage of β-lactamase and carbapenemase-producing Enterobacteriaceae in hospitalized patients at debre tabor comprehensive specialized hospital. *Heliyon*. 2023;**9**(9):e20072.10.1016/j.heliyon.2023.e20072.

7. Amare A, Eshetie S, Kasew D, Moges F. High prevalence of fecal carriage of Extended-spectrum beta-lactamase and carbapenemase-producing Enterobacteriaceae among food handlers at the University of Gondar, Northwest Ethiopia. *PloS one*. 2022;**17**(3):e0264818.10.1371/journal.pone.0264818.

8. Diriba K, Awulachew E, 2nd, Tekele L, Ashuro Z. Fecal Carriage Rate of Extended-Spectrum Beta-Lactamase-Producing Escherichia coli and Klebsiella pneumoniae Among Apparently Health Food Handlers in Dilla University Student Cafeteria. *Infection and drug resistance*. 2020;**13**:3791-800.10.2147/idr.S269425.

9. Aklilu A, Manilal A, Ameya G, Woldemariam M, Siraj M. Gastrointestinal Tract Colonization Rate of Extended﻿-Spectrum Beta-Lactamase- and Carbapenemase-Producing Enterobacteriaceae and Associated Factors Among Hospitalized Patients in Arba Minch General Hospital, Arba Minch, Ethiopia. *Infection and drug resistance*. 2020;**13**:1517-26.10.2147/idr.S239092.

10. Bayleyegn B, Fisaha R, Kasew D. Fecal carriage of extended spectrum beta-lactamase producing Enterobacteriaceae among HIV infected children at the University of Gondar Comprehensive Specialized Hospital Gondar, Ethiopia. *AIDS research and therapy*. 2021;**18**(1):19.10.1186/s12981-021-00347-x.

11. Worku M, Getie M, Moges F, Mehari AG. Extended-Spectrum Beta-Lactamase- and Carbapenemase-Producing Enterobacteriaceae Family of Bacteria from Diarrheal Stool Samples in Northwest Ethiopia. *Interdisciplinary perspectives on infectious diseases*. 2022;**2022**:7905350.10.1155/2022/7905350.

12. Desta K, Woldeamanuel Y, Azazh A, Mohammod H, Desalegn D, Shimelis D, et al. High Gastrointestinal Colonization Rate with Extended-Spectrum beta-Lactamase-Producing Enterobacteriaceae in Hospitalized Patients: Emergence of Carbapenemase-Producing K. pneumoniae in Ethiopia. *PLoS One*. 2016;**11**(8):e0161685.10.1371/journal.pone.0161685.

13. Wolde D, Eguale T, Alemayehu H, Medhin G, Haile AF, Pirs M, et al. Antimicrobial Susceptibility and Characterization of Extended-Spectrum β-Lactamase-Producing Escherichia coli Isolated from Stools of Primary Healthcare Patients in Ethiopia. *Antibiotics* 2024;**13**(1).10.3390/antibiotics13010093.

14. Yonas Mekonnen SS, Alganesh Gebreyohanns, Dejenie Shiferaw Teklu, Zeleke Ayenew, Amete Mihret, Zelalem Tazu Bonger. Fecal Carriage of Carbapenem-resistant Enterobacterales and Associated Factors Among Admitted Patients in Saint Paul’s Hospital Millennium Medical College, Addis Ababa, Ethiopia. *Infection and drug resistance*. 2023;**16**:6345–55

15. Amsalu G, Wen CT, Perovic O, Gebru A, Hunegnaw BM, Tadesse F, et al. Carriage of antimicrobial-resistant Enterobacterales among pregnant women and newborns in Amhara, Ethiopia. *International Journal of Infectious Diseases*. 2024;**143**:107035.10.1016/j.ijid.2024.107035.

16. Campos-Madueno EI, Moradi M, Eddoubaji Y, Shahi F, Moradi S, Bernasconi OJ, et al. Intestinal colonization with multidrug-resistant Enterobacterales: screening, epidemiology, clinical impact, and strategies to decolonize carriers. *Eur J Clin Microbiol Infect Dis*. 2023;**42**(3):229-54.10.1007/s10096-023-04548-2.

17. Nakai M, Oka K, Watanabe G, Kamei K, Tsukada N, Mori R, et al. Epidemiology and molecular characterization of fecal carriage of third-generation cephalosporin-resistant Enterobacterales among elderly residents in Japan. *J Infect Chemother*. 2022;**28**(4):569-75.10.1016/j.jiac.2021.12.033.

18. Habibzadeh N, Peeri Doghaheh H, Manouchehri Far M, Alimohammadi Asl H, Iranpour S, Arzanlou M. Fecal Carriage of Extended-Spectrum β-Lactamases and pAmpC Producing Enterobacterales in an Iranian Community: Prevalence, Risk Factors, Molecular Epidemiology, and Antibiotic Resistance. *Microb Drug Resist*. 2022;**28**(9):921-34.10.1089/mdr.2021.0029.

19. El Aila NA, Laham NAA, Ayesh BM, Naas T. Fecal carriage of extended-spectrum β-lactamase-producing enterobacterales from hospitals and community settings in Gaza Strip, Palestine. *BMC Microbiol*. 2023;**23**(1):376.10.1186/s12866-023-03102-6.

20. Promsuwan O, Malathum K, Ingsathit A. Epidemiology of extended-spectrum β-lactamase-producing Enterobacterales infection in kidney transplant recipients. *Antimicrob Resist Infect Control*. 2023;**12**(1):123.10.1186/s13756-023-01308-x.

21. Pérez-Nadales E, Fernández-Ruiz M, Gutiérrez-Gutiérrez B, Pascual Á, Rodríguez-Baño J, Martínez-Martínez L, et al. Extended-spectrum β-lactamase-producing and carbapenem-resistant Enterobacterales bloodstream infection after solid organ transplantation: Recent trends in epidemiology and therapeutic approaches. *Transpl Infect Dis*. 2022;**24**(4):e13881.10.1111/tid.13881.

22. Moxon CA, Paulus S. Beta-lactamases in Enterobacteriaceae infections in children. *J Infect*. 2016;**72 Suppl**:S41-9.10.1016/j.jinf.2016.04.021.

23. Jiménez-Rojas V, Villanueva-García D, Miranda-Vega AL, Aldana-Vergara R, Aguilar-Rodea P, López-Marceliano B, et al. Gut colonization and subsequent infection of neonates caused by extended-spectrum beta-lactamase-producing Escherichia coli and Klebsiella pneumoniae. *Front Cell Infect Microbiol*. 2023;**13**:1322874.10.3389/fcimb.2023.1322874.

24. Jacquier H, Assao B, Chau F, Guindo O, Condamine B, Magnan M, et al. Faecal carriage of extended-spectrum β-lactamase-producing Escherichia coli in a remote region of Niger. *J Infect*. 2023;**87**(3):199-209.10.1016/j.jinf.2023.06.015.

25. Armand-Lefèvre L, Rondinaud E, Desvillechabrol D, Mullaert J, Clermont O, Petitjean M, et al. Dynamics of extended-spectrum beta-lactamase-producing Enterobacterales colonization in long-term carriers following travel abroad. *Microb Genom*. 2021;**7**(7).10.1099/mgen.0.000576.

26. Ruppé E, Woerther PL, Diop A, Sene AM, Da Costa A, Arlet G, et al. Carriage of CTX-M-15-producing Escherichia coli isolates among children living in a remote village in Senegal. *Antimicrob Agents Chemother*. 2009;**53**(7):3135-7.10.1128/aac.00139-09.

27. Chuang C, Lee KC, Wang YP, Lee PC, Chang TE, Huang YH, et al. High carriage rate of extended-spectrum β-lactamase Enterobacterales and diarrheagenic Escherichia coli in healthy donor screening for fecal microbiota transplantation. *Eur J Clin Microbiol Infect Dis*. 2023;**42**(9):1103-13.10.1007/s10096-023-04644-3.

28. Ahmed SF, Ali MM, Mohamed ZK, Moussa TA, Klena JD. Fecal carriage of extended-spectrum β-lactamases and AmpC-producing Escherichia coli in a Libyan community. *Ann Clin Microbiol Antimicrob*. 2014;**13**:22.10.1186/1476-0711-13-22.

29. Obeng-Nkrumah N, Hansen DS, Awuah-Mensah G, Blankson NK, Frimodt-Møller N, Newman MJ, et al. High level of colonization with third-generation cephalosporin-resistant Enterobacterales in African community settings, Ghana. *Diagn Microbiol Infect Dis*. 2023;**106**(1):115918.10.1016/j.diagmicrobio.2023.115918.

30. Woerther PL, Burdet C, Chachaty E, Andremont A. Trends in human fecal carriage of extended-spectrum β-lactamases in the community: toward the globalization of CTX-M. *Clin Microbiol Rev*. 2013;**26**(4):744-58.10.1128/cmr.00023-13.

31. Ding Y, Saw WY, Tan LWL, Moong DKN, Nagarajan N, Teo YY, et al. Extended-Spectrum β-Lactamase-Producing and mcr-1-Positive Escherichia coli from the Gut Microbiota of Healthy Singaporeans. *Appl Environ Microbiol*. 2021;**87**(20):e0048821.10.1128/aem.00488-21.

32. Barani A, Tabatabaee Bafroee AS, Jabalameli L. Abundance of extended-spectrum β-lactamase genes among intestinal Escherichia coli strains from drug users. *Arch Microbiol*. 2021;**203**(6):3245-55.10.1007/s00203-021-02316-4.

33. Ford CD, Lopansri BK, Coombs J, Gouw L, Asch J, Hoda D. Extended spectrum cephalosporin resistant enterobacteriaceae carriage and infection in patients admitted with newly-diagnosed acute leukemia. *Am J Infect Control*. 2023;**51**(2):172-7.10.1016/j.ajic.2022.05.019.

34. Pérez-López A, Sundararaju S, Tsui KM, Al-Mana H, Hasan MR, Suleiman M, et al. Fecal Carriage and Molecular Characterization of Carbapenemase-Producing Enterobacterales in the Pediatric Population in Qatar. *Microbiol Spectr*. 2021;**9**(3):e0112221.10.1128/Spectrum.01122-21.

35. Aires-de-Sousa M, Lopes E, Gonçalves ML, Pereira AL, Machado ECA, de Lencastre H, et al. Intestinal carriage of extended-spectrum beta-lactamase-producing Enterobacteriaceae at admission in a Portuguese hospital. *Eur J Clin Microbiol Infect Dis*. 2020;**39**(4):783-90.10.1007/s10096-019-03798-3.

36. Ouchar Mahamat O, Tidjani A, Lounnas M, Hide M, Benavides J, Somasse C, et al. Fecal carriage of extended-spectrum β-lactamase-producing Enterobacteriaceae in hospital and community settings in Chad. *Antimicrob Resist Infect Control*. 2019;**8**:169.10.1186/s13756-019-0626-z.

37. Huang IF, Lee WY, Wang JL, Hung CH, Hu HH, Hung WY, et al. Fecal carriage of multidrug-resistant Escherichia coli by community children in southern Taiwan. *BMC Gastroenterol*. 2018;**18**(1):86.10.1186/s12876-018-0807-x.

38. Hammami S, Dahdeh C, Mamlouk K, Ferjeni S, Maamar E, Hamzaoui Z, et al. Rectal Carriage of Extended-Spectrum Beta-Lactamase and Carbapenemase Producing Gram-Negative Bacilli in Intensive Care Units in Tunisia. *Microb Drug Resist*. 2017;**23**(6):695-702.10.1089/mdr.2016.0205.

39. Hajihasani A, Ebrahimi-Rad M, Rasoulinasab M, Aslani MM, Shahcheraghi F. Prevalence of O25b-ST131 Escherichia coli Clone: Fecal Carriage of Extended-Spectrum β-Lactamase and Carbapenemase-Producing Isolates in Healthy Adults in Tehran, Iran. *Microb Drug Resist*. 2022;**28**(2):210-6.10.1089/mdr.2021.0001.

40. Ferjani S, Saidani M, Hamzaoui Z, Alonso CA, Torres C, Maamar E, et al. Community fecal carriage of broad-spectrum cephalosporin-resistant Escherichia coli in Tunisian children. *Diagn Microbiol Infect Dis*. 2017;**87**(2):188-92.10.1016/j.diagmicrobio.2016.03.008.

41. Kawata S, Morimoto S, Kosai K, Kawamoto Y, Nakashima Y, Morinaga Y, et al. The fecal carriage rate of extended-spectrum β-lactamase-producing or carbapenem-resistant Enterobacterales among Japanese infants in the community at the 4-month health examination in a rural city. *Front Cell Infect Microbiol*. 2023;**13**:1168451.10.3389/fcimb.2023.1168451.

42. Akenten CW, Khan NA, Mbwana J, Krumkamp R, Fosu D, Paintsil EK, et al. Carriage of ESBL-producing Klebsiella pneumoniae and Escherichia coli among children in rural Ghana: a cross-sectional study. *Antimicrob Resist Infect Control*. 2023;**12**(1):60.10.1186/s13756-023-01263-7.

43. Grall-Zahar I, Rucly S, Billard-Pomares T, Gasnier-Besnardeau K, Al Mouft O, Zahar JR, et al. Prevalence and risk factors for carriage of extended-spectrum β-lactamase-producing enterobacteriaceae in rehabilitation wards in France. *Infect Dis Now*. 2022;**52**(7):403-7.10.1016/j.idnow.2022.07.004.

44. Barguigua A, Ouair H, El Otmani F, Saile R, El Mdaghri N, El Azhari M, et al. Fecal carriage of extended-spectrum β-lactamase-producing Enterobacteriaceae in community setting in Casablanca. *Infect Dis (Lond)*. 2015;**47**(1):27-32.10.3109/00365548.2014.961542.

45. Çakir Erdoğan D, Cömert F, Aktaş E, Köktürk F, Külah C. Fecal carriage of extended-spectrum beta-lactamase-producing Escherichia coli and Klebsiella spp. in a Turkish community. *Turk J Med Sci*. 2017;**47**(1):172-9.10.3906/sag-1512-9.

46. Bassyouni RH, Gaber SN, Wegdan AA. Fecal carriage of extended-spectrum β-lactamase- and AmpC- producing Escherichia coli among healthcare workers. *J Infect Dev Ctries*. 2015;**9**(3):304-8.10.3855/jidc.5633.

47. Pan F, Tian D, Wang B, Zhao W, Qin H, Zhang T, et al. Fecal carriage and molecular epidemiology of carbapenem-resistant Enterobacteriaceae from outpatient children in Shanghai. *BMC Infect Dis*. 2019;**19**(1):678.10.1186/s12879-019-4298-3.

48. Denkel LA, Maechler F, Schwab F, Kola A, Weber A, Gastmeier P, et al. Infections caused by extended-spectrum β-lactamase-producing Enterobacterales after rectal colonization with ESBL-producing Escherichia coli or Klebsiella pneumoniae. *Clin Microbiol Infect*. 2020;**26**(8):1046-51.10.1016/j.cmi.2019.11.025.

49. Dandachi I, Salem Sokhn E, Najem E, Azar E, Daoud Z. Carriage of beta-lactamase-producing Enterobacteriaceae among nursing home residents in north Lebanon. *Int J Infect Dis*. 2016;**45**:24-31.10.1016/j.ijid.2016.02.007.

50. Dimani BD, Founou RC, Zemtsa JR, Mbossi A, Koudoum PL, Founou LL, et al. Faecal carriage of multidrug-resistant and extended-spectrum β-lactamase-producing Enterobacterales in people living with HIV in Yaoundé, Cameroon. *J Glob Antimicrob Resist*. 2023;**35**:26-34.10.1016/j.jgar.2023.07.021.

51. Aghamohammad S, Nikbin VS, Badmasti F, Shahcheraghi F. High heterogeneity of fecal carriage extended-spectrum beta-lactamase-producing E. coli isolated from iranian community and clinical settings. *BMC Infect Dis*. 2022;**22**(1):318.10.1186/s12879-022-07304-7.

52. Kibwana UO, Manyahi J, Sandnes HH, Blomberg B, Mshana SE, Langeland N, et al. Gastrointestinal colonization of extended-spectrum beta-lactamase-producing bacteria among children below five years of age hospitalized with fever in Dar es Salaam, Tanzania. *J Glob Antimicrob Resist*. 2022;**30**:107-14.10.1016/j.jgar.2022.05.023.

53. Mathai D, Kumar VA, Paul B, Sugumar M, John KR, Manoharan A, et al. Fecal carriage rates of extended-spectrum β-lactamase-producing Escherichia coli among antibiotic naive healthy human volunteers. *Microb Drug Resist*. 2015;**21**(1):59-64.10.1089/mdr.2014.0031.

54. Ducarmon QR, Zwittink RD, Willems RPJ, Verhoeven A, Nooij S, van der Klis FRM, et al. Gut colonisation by extended-spectrum β-lactamase-producing Escherichia coli and its association with the gut microbiome and metabolome in Dutch adults: a matched case-control study. *Lancet Microbe*. 2022;**3**(6):e443-e51.10.1016/s2666-5247(22)00037-4.

55. Jolivet S, Vaillant L, Poncin T, Lolom I, Gaudonnet Y, Rondinaud E, et al. Prevalence of carriage of extended-spectrum β-lactamase-producing enterobacteria and associated factors in a French hospital. *Clin Microbiol Infect*. 2018;**24**(12):1311-4.10.1016/j.cmi.2018.03.008.

56. Al-Agamy MH, El Mahdy TS, Shibl AM. Fecal Colonization with Extended-Spectrum Beta-Lactamase and AmpC-Producing Escherichia coli. *Biomed Res Int*. 2016;**2016**:3704150.10.1155/2016/3704150.

57. Golzarri MF, Silva-Sánchez J, Cornejo-Juárez P, Barrios-Camacho H, Chora-Hernández LD, Velázquez-Acosta C, et al. Colonization by fecal extended-spectrum β-lactamase-producing Enterobacteriaceae and surgical site infections in patients with cancer undergoing gastrointestinal and gynecologic surgery. *Am J Infect Control*. 2019;**47**(8):916-21.10.1016/j.ajic.2019.01.020.

58. Kibwana UO, Majigo M, Kamori D, Manyahi J. High fecal carriage of extended Beta Lactamase producing Enterobacteriaceae among adult patients admitted in referral hospitals in Dar es Salaam, Tanzania. *BMC Infect Dis*. 2020;**20**(1):557.10.1186/s12879-020-05272-4.

59. Alelign D, Kidanewold A. Magnitude of extended-spectrum β-lactamase and carbapenemase producing Enterobacteriaceae among commonly vended street foods in Arba Minch town, southern Ethiopia. *BMC Microbiol*. 2023;**23**(1):393.10.1186/s12866-023-03137-9.

60. Woldeteklie AA, Kebede HB, Abdela AA, Woldeamanuel Y. Prevalence of Extended-Spectrum β-Lactamase and Carbapenemase Producers of Gram-Negative Bacteria, and Methicillin-Resistant Staphylococcus aureus in Isolates from Diabetic Foot Ulcer Patients in Ethiopia. *Infect Drug Resist*. 2022;**15**:4435-41.10.2147/idr.S371431.

61. Worku S, Abebe T, Seyoum B, Alemu B, Denkayehu G, Seyoum T, et al. Molecular characterization of carbapenemase and extended spectrum beta-lactamase producing Acinetobacter baumannii isolates causing surgical site infections in Ethiopia. *BMC Infect Dis*. 2024;**24**(1):459.10.1186/s12879-024-09362-5.

62. Tilahun M, Gedefie A, Bisetegn H, Debash H. Emergence of High Prevalence of Extended-Spectrum Beta-Lactamase and Carbapenemase Producing Acinetobacter Species and Pseudomonas aeruginosa Among Hospitalized Patients at Dessie Comprehensive Specialized Hospital, North-East Ethiopia. *Infect Drug Resist*. 2022;**15**:895-911.10.2147/idr.S358116.

63. Tigabie M, Biset S, Belachew T, Amare A, Moges F. Multidrug-resistant and extended-spectrum beta-lactamase-producing Enterobacteriaceae isolated from chicken droppings in poultry farms at Gondar City, Northwest Ethiopia. *PLoS One*. 2023;**18**(6):e0287043.10.1371/journal.pone.0287043.

64. Desalegn Y, Bitew A, Adane A. A spectrum of non-spore-forming fermentative and non-fermentative Gram-negative bacteria: multi-drug resistance, extended-spectrum beta-lactamase, and carbapenemase production. *Front Antibiot*. 2023;**2**:1155005.10.3389/frabi.2023.1155005.

65. Engda T, Moges F, Gelaw A, Eshete S, Mekonnen F. Prevalence and antimicrobial susceptibility patterns of extended spectrum beta-lactamase producing Entrobacteriaceae in the University of Gondar Referral Hospital environments, northwest Ethiopia. *BMC Res Notes*. 2018;**11**(1):335.10.1186/s13104-018-3443-1.

66. Kebede AA, Bedada TL, Teklu DS, Beyene D, Tullu KD. Occurrence and anti-microbial susceptibility pattern of extended spectrum beta-lactamase producing Enterobacteriaceae in governmental hospitals wastewater in Addis Ababa, Ethiopia. *Trop Med Health*. 2022;**50**(1):57.10.1186/s41182-022-00437-0.

67. Abayneh M, Tesfaw G, Woldemichael K, Yohannis M, Abdissa A. Assessment of extended-spectrum β-lactamase (ESBLs) - producing Escherichia coli from minced meat of cattle and swab samples and hygienic status of meat retailer shops in Jimma town, Southwest Ethiopia. *BMC Infect Dis*. 2019;**19**(1):897.10.1186/s12879-019-4554-6.

68. Worku W, Desta M, Menjetta T. High prevalence and antimicrobial susceptibility pattern of salmonella species and extended-spectrum β-lactamase producing Escherichia coli from raw cattle meat at butcher houses in Hawassa city, Sidama regional state, Ethiopia. *PLoS One*. 2022;**17**(1):e0262308.10.1371/journal.pone.0262308.

69. Asfaw T, Genetu D, Shenkute D, Shenkutie TT, Amare YE, Yitayew B. High Levels of Multidrug-Resistant and Beta-Lactamase-Producing Bacteria in Meat and Meat Contact Surfaces, Debre Berhan Town, Ethiopia. *Infect Drug Resist*. 2023;**16**:1965-77.10.2147/idr.S405582.

70. Nigussie D, Amsalu A. Prevalence of uropathogen and their antibiotic resistance pattern among diabetic patients. *Turk J Urol*. 2017;**43**(1):85-92.10.5152/tud.2016.86155.

71. Reda BK, Molla G, Gedefie A, Gebretsadik D, Tilahun M, Belete MA, et al. Antibiogram of uropathogens and associated risk factors among asymptomatic female college students in Dessie town, Northeast Ethiopia. *PLoS One*. 2023;**18**(11):e0276033.10.1371/journal.pone.0276033.

72. Mussema A, Admasu D, Bawore S, Abdo R, Seid A. BACTERIAL PROFILE, ANTIMICROBIAL RESISTANCE, AND FACTORS ASSOCIATED WITH URINARY TRACT INFECTION AMONG PREGNANT WOMEN AT HOSANNA TOWN HEALTH FACILITIES, CENTRAL ETHIOPIA. *Georgian Med News*. 2023(342):113-21

73. Usmael B, Abraha B, Alemu S, Mummed B, Hiko A, Abdurehman A. Isolation, antimicrobial susceptibility patterns, and risk factors assessment of non-typhoidal Salmonella from apparently healthy and diarrheic dogs. *BMC Vet Res*. 2022;**18**(1):37.10.1186/s12917-021-03135-x.

74. Tadesse S, Kahsay T, Adhanom G, Kahsu G, Legese H, A GW, et al. Prevalence, antimicrobial susceptibility profile and predictors of asymptomatic bacteriuria among pregnant women in Adigrat General Hospital, Northern Ethiopia. *BMC Res Notes*. 2018;**11**(1):740.10.1186/s13104-018-3844-1.

75. Abu D, Abula T, Zewdu T, Berhanu M, Sahilu T. Asymptomatic Bacteriuria, antimicrobial susceptibility pattern and associated risk factors among pregnant women attending antenatal care in Assosa General Hospital, Western Ethiopia. *BMC Microbiol*. 2021;**21**(1):348.10.1186/s12866-021-02417-6.

76. Marami D, Hailu K, Tolera M. Prevalence and antimicrobial susceptibility pattern of Salmonella and Shigella species among asymptomatic food handlers working in Haramaya University cafeterias, Eastern Ethiopia. *BMC Res Notes*. 2018;**11**(1):74.10.1186/s13104-018-3189-9.

77. Legese H, Kahsay T, Gebrewahd A, Berhe B, Fseha B, Tadesse S, et al. Prevalence, antimicrobial susceptibility pattern, and associated factors of Salmonella and Shigella among food handlers in Adigrat University student's cafeteria, northern Ethiopia, 2018. *Trop Dis Travel Med Vaccines*. 2020;**6**:19.10.1186/s40794-020-00119-x.

78. Mama M, Alemu G. Prevalence, antimicrobial susceptibility patterns and associated risk factors of Shigella and Salmonella among food handlers in Arba Minch University, South Ethiopia. *BMC Infect Dis*. 2016;**16**(1):686.10.1186/s12879-016-2035-8.

79. Beyi AF, Fite AT, Tora E, Tafese A, Genu T, Kaba T, et al. Prevalence and antimicrobial susceptibility of Escherichia coli O157 in beef at butcher shops and restaurants in central Ethiopia. *BMC Microbiol*. 2017;**17**(1):49.10.1186/s12866-017-0964-z.

80. Gutema FD, Rasschaert G, Agga GE, Jufare A, Duguma AB, Abdi RD, et al. Occurrence, Molecular Characteristics, and Antimicrobial Resistance of Escherichia coli O157 in Cattle, Beef, and Humans in Bishoftu Town, Central Ethiopia. *Foodborne Pathog Dis*. 2021;**18**(1):1-7.10.1089/fpd.2020.2830.

81. Assefa A, Asrat D, Woldeamanuel Y, Y GH, Abdella A, Melesse T. Bacterial profile and drug susceptibility pattern of urinary tract infection in pregnant women at Tikur Anbessa Specialized Hospital Addis Ababa, Ethiopia. *Ethiop Med J*. 2008;**46**(3):227-35

82. Tigabu A, Ferede W, Belay G, Gelaw B. Prevalence of Asymptomatic Bacteriuria and Antibiotic Susceptibility Patterns of Bacterial Isolates among Cancer Patients and Healthy Blood Donors at the University of Gondar Specialized Hospital. *Int J Microbiol*. 2020;**2020**:3091564.10.1155/2020/3091564.

83. Belyhun Y, Moges F, Endris M, Asmare B, Amare B, Bekele D, et al. Ocular bacterial infections and antibiotic resistance patterns in patients attending Gondar Teaching Hospital, Northwest Ethiopia. *BMC Res Notes*. 2018;**11**(1):597.10.1186/s13104-018-3705-y.

84. Feleke T, Eshetie S, Dagnew M, Endris M, Abebe W, Tiruneh M, et al. Multidrug-resistant bacterial isolates from patients suspected of nosocomial infections at the University of Gondar Comprehensive Specialized Hospital, Northwest Ethiopia. *BMC Res Notes*. 2018;**11**(1):602.10.1186/s13104-018-3709-7.

85. Million Y, Feleke T, Mengesha D, Senay B, Tigabu A. Multidrug-Resistant Bacteria Among Culture Isolates at University of Gondar, Specialized Referral Hospital, Northwest Ethiopia: a Five-Year Retrospective Study. *Clin Lab*. 2020;**66**(7).10.7754/Clin.Lab.2019.190941.

86. Muluye D, Wondimeneh Y, Ferede G, Moges F, Nega T. Bacterial isolates and drug susceptibility patterns of ear discharge from patients with ear infection at Gondar University Hospital, Northwest Ethiopia. *BMC Ear Nose Throat Disord*. 2013;**13**(1):10.10.1186/1472-6815-13-10.

87. Wasihun AG, Zemene Y. Bacterial profile and antimicrobial susceptibility patterns of otitis media in Ayder Teaching and Referral Hospital, Mekelle University, Northern Ethiopia. *Springerplus*. 2015;**4**:701.10.1186/s40064-015-1471-z.

88. Worku S, Gelaw A, Aberra Y, Muluye D, Derbie A, Biadglegne F. Bacterial etiologies, antibiotic susceptibility patterns and risk factors among patients with ear discharge at the University of Gondar Hospital, Northwest Ethiopia. *Asian Pac J Trop Dis*. 2017;**7**(1):36-42

89. Hailu D, Mekonnen D, Derbie A, Mulu W, Abera B. Pathogenic bacteria profile and antimicrobial susceptibility patterns of ear infection at Bahir Dar Regional Health Research Laboratory Center, Ethiopia. *Springerplus*. 2016;**5**:466.10.1186/s40064-016-2123-7.

90. Hailegiyorgis TT, Sarhie WD, Workie HM. Isolation and antimicrobial drug susceptibility pattern of bacterial pathogens from pediatric patients with otitis media in selected health institutions, Addis Ababa, Ethiopia: a prospective cross-sectional study. *BMC Ear Nose Throat Disord*. 2018;**18**:8.10.1186/s12901-018-0056-1.

91. Gorems K, Beyene G, Berhane M, Mekonnen Z. Antimicrobial susceptibility patterns of bacteria isolated from patients with ear discharge in Jimma Town, Southwest, Ethiopia. *BMC Ear Nose Throat Disord*. 2018;**18**:17.10.1186/s12901-018-0065-0.

92. Molla R, Tiruneh M, Abebe W, Moges F. Bacterial profile and antimicrobial susceptibility patterns in chronic suppurative otitis media at the University of Gondar Comprehensive Specialized Hospital, Northwest Ethiopia. *BMC Res Notes*. 2019;**12**(1):414.10.1186/s13104-019-4452-4.

93. Tadesse B, Shimelis T, Worku M. Bacterial profile and antibacterial susceptibility of otitis media among pediatric patients in Hawassa, Southern Ethiopia: cross-sectional study. *BMC Pediatr*. 2019;**19**(1):398.10.1186/s12887-019-1781-3.

94. Haile Z, Mengist HM, Dilnessa T. Bacterial isolates, their antimicrobial susceptibility pattern, and associated factors of external ocular infections among patients attending eye clinic at Debre Markos Comprehensive Specialized Hospital, Northwest Ethiopia. *PLoS One*. 2022;**17**(11):e0277230.10.1371/journal.pone.0277230.

95. Flokas ME, Alevizakos M, Shehadeh F, Andreatos N, Mylonakis E. Extended-spectrum β-lactamase-producing Enterobacteriaceae colonisation in long-term care facilities: a systematic review and meta-analysis. *Int J Antimicrob Agents*. 2017;**50**(5):649-56.10.1016/j.ijantimicag.2017.08.003.

96. Alevizakos M, Karanika S, Detsis M, Mylonakis E. Colonisation with extended-spectrum β-lactamase-producing Enterobacteriaceae and risk for infection among patients with solid or haematological malignancy: a systematic review and meta-analysis. *Int J Antimicrob Agents*. 2016;**48**(6):647-54.10.1016/j.ijantimicag.2016.08.021.

97. Abera D, Alemu A, Mihret A, Negash AA, Abegaz WE, Cadwell KJPo. Colonization with extended spectrum beta-lactamase and carbapenemases producing Enterobacteriaceae among hospitalized patients at the global level: A systematic review and meta-analysis. 2023;**18**(11):e0293528

98. Kiros T, Workineh L, Tiruneh T, Eyayu T, Damtie S, Belete DJIjom. Prevalence of Extended‐Spectrum β‐Lactamase‐Producing Enterobacteriaceae in Ethiopia: A Systematic Review and Meta‐Analysis. 2021;**2021**(1):6669778

99. Diriba K, Awulachew E, Gemede A, Anja AJAM. The magnitude of extended-spectrum beta-lactamase-producing Enterobacteriaceae from clinical samples in Ethiopia: a systematic review and meta-analysis. 2021;**3**(3):000195

100. Tufa TB, Fuchs A, Tufa TB, Stötter L, Kaasch AJ, Feldt T, et al. High rate of extended-spectrum beta-lactamase-producing gram-negative infections and associated mortality in Ethiopia: a systematic review and meta-analysis. 2020;**9**:1-10

101. Lewis JM, Lester R, Garner P, Feasey NAJWor. Gut mucosal colonisation with extended-spectrum beta-lactamase producing Enterobacteriaceae in sub-Saharan Africa: a systematic review and meta-analysis. 2020;**4**:160

102. Alemayehu E, Fiseha T, Gedefie A, Alemayehu Tesfaye N, Ebrahim H, Ebrahim E, et al. Prevalence of carbapenemase-producing Enterobacteriaceae from human clinical samples in Ethiopia: a systematic review and meta-analysis. 2023;**23**(1):277

103. Abayneh M, Worku TJDTI. Prevalence of multidrug-resistant and extended-spectrum beta-lactamase (ESBL)-producing gram-negative bacilli: a meta-analysis report in Ethiopia. 2020;**14**:16

104. Sonda T, Kumburu H, van Zwetselaar M, Alifrangis M, Lund O, Kibiki G, et al. Meta-analysis of proportion estimates of Extended-Spectrum-Beta-Lactamase-producing Enterobacteriaceae in East Africa hospitals. 2016;**5**:1-9

105. Jalilian N, Kooshkiforooshani M, Ahmadi S, Nankali AJJoGAR. Colonisation with extended-spectrum β-lactamase-producing Enterobacteriaceae in pregnant/post-partum women: Systematic review and meta-analysis. 2019;**19**:338-47

106. Timbrook TT, Fowler MJJA. Predicting extended-spectrum beta-lactamase and carbapenem resistance in Enterobacteriaceae bacteremia: a diagnostic model systematic review and meta-analysis. 2023;**12**(9):1452

107. Tesfa T, Mitiku H, Edae M, Assefa NJSr. Prevalence and incidence of carbapenem-resistant K. pneumoniae colonization: systematic review and meta-analysis. 2022;**11**(1):240

108. Caliskan-Aydogan O, Alocilja ECJM. A review of carbapenem resistance in Enterobacterales and its detection techniques. 2023;**11**(6):1491

109. Legese MH, Asrat D, Aseffa A, Hasan B, Mihret A, Swedberg GJA. Molecular epidemiology of extended-spectrum beta-lactamase and AmpC producing Enterobacteriaceae among sepsis patients in Ethiopia: a prospective multicenter study. 2022;**11**(2):131

110. Alebel M, Mekonnen F, Mulu WJI, Resistance D. Extended-spectrum β-lactamase and carbapenemase producing gram-negative bacilli infections among patients in intensive care units of felegehiwot referral hospital: a prospective cross-sectional study. 2021:391-405

111. Abayneh M, Tesfaw G, Abdissa A. Isolation of Extended-Spectrum beta-lactamase- (ESBL-) Producing Escherichia coli and Klebsiella pneumoniae from Patients with Community-Onset Urinary Tract Infections in Jimma University Specialized Hospital, Southwest Ethiopia. *Can J Infect Dis Med Microbiol*. 2018;**2018**:4846159.10.1155/2018/4846159.

112. Zeleke Gizachew TK, Getnet Beyene, Rawleigh Howe, Biruk Yeshitila. . Multi-drug resistant bacteria and associated factors among reproductive age women with significant bacteriuria. *Ethiopian Medical Journal*. 2019;**573**:31-43

113. Gebremariam G, Legese H, Woldu Y, Araya T, Hagos K, GebreyesusWasihun A. Bacteriological profile, risk factors and antimicrobial susceptibility patterns of symptomatic urinary tract infection among students of Mekelle University, northern Ethiopia. *BMC Infect Dis*. 2019;**19**(1):950.10.1186/s12879-019-4610-2.

114. Eshetie S, Balew M, Mekonnen F, Biset S, Amsalu A, Endalamaw D. Extended-Spectrum β-Lactamases-Producing Enterobacteria and Antimicrobial Resistance Pattern among HIV/AIDS Patients in the University of Gondar Specialized Hospital, Ethiopia. *Recent Advances in Biology and Medicine*. 2020;**6**.10.18639/rabm.2020.964046.

115. Biset S, Moges F, Endalamaw D, Eshetie S. Multi-drug resistant and extended-spectrum beta-lactamases producing bacterial uropathogens among pregnant women in Northwest Ethiopia. *Annals of Clinical Microbiology and Antimicrobials*. 2020;**19**(1):25.10.1186/s12941-020-00365-z.

116. Belete MA. Bacterial Profile and ESBL Screening of Urinary Tract Infection Among Asymptomatic and Symptomatic Pregnant Women Attending Antenatal Care of Northeastern Ethiopia Region. *Infect Drug Resist*. 2020;**13**:2579-92.10.2147/IDR.S258379.

117. Alemu M, Belete MA, Gebreselassie S, Belay A, Gebretsadik D. Bacterial Profiles and Their Associated Factors of Urinary Tract Infection and Detection of Extended Spectrum Beta-Lactamase Producing Gram-Negative Uropathogens Among Patients with Diabetes Mellitus at Dessie Referral Hospital, Northeastern Ethiopia. *Diabetes Metab Syndr Obes*. 2020;**13**:2935-48.10.2147/DMSO.S262760.

118. Agegnehu A, Worku M, Nigussie D, Lulu B, Tadesse BT. Pediatric Febrile Urinary Tract Infection Caused by ESBL Producing Enterobacteriaceae Species. *Biomed Res Int*. 2020;**2020**:6679029.10.1155/2020/6679029.

119. Getie M, Gebre-Selassie S, Getu Y, Birara S, Tiruneh C, Abebaw A, et al. Bacterial profile and extended spectrum beta lactamase screening of urinary tract infection among asymptomatic and symptomatic pregnant women attending antenatal care in ALERT Hospital, Addis Ababa, Ethiopia. *SAGE Open Med*. 2023;**11**:20503121231197587.10.1177/20503121231197587.

120. Fenta A, Dagnew M, Eshetie S, Belachew T. Bacterial profile, antibiotic susceptibility pattern and associated risk factors of urinary tract infection among clinically suspected children attending at Felege-Hiwot comprehensive and specialized hospital, Northwest Ethiopia. A prospective study. *BMC Infect Dis*. 2020;**20**(1):673.10.1186/s12879-020-05402-y.

121. Kasew D, Eshetie S, Diress A, Tegegne Z, Moges F. Multiple drug resistance bacterial isolates and associated factors among urinary stone patients at the University of Gondar Comprehensive Specialized Hospital, Northwest Ethiopia. *BMC Urol*. 2021;**21**(1):27.10.1186/s12894-021-00794-8.

122. Ameshe A, Engda T, Gizachew M. Antimicrobial Resistance Patterns, Extended-Spectrum Beta-Lactamase Production, and Associated Risk Factors of Klebsiella Species among UTI-Suspected Patients at Bahir Dar City, Northwest Ethiopia. *Int J Microbiol*. 2022;**2022**:8216545.10.1155/2022/8216545.

123. Gebremedhin MG, Weldu Y, Kahsay AG, Teame G, Adane K. Extended-Spectrum beta-Lactamase and Carbapenemase-Producing Gram-Negative Bacteria and Associated Factors Among Patients Suspected of Community and Hospital-Acquired Urinary Tract Infections at Ayder Comprehensive Specialized Hospital, Tigrai, Ethiopia. *Infect Drug Resist*. 2023;**16**:4025-37.10.2147/IDR.S412350.

124. Elale AK, Manilal A, Tadesse D, Seid M, Dubale A. Magnitude and associated factors of bacterial urinary tract infections among paediatric patients in Arba Minch, southern Ethiopia. *New Microbes New Infect*. 2023;**51**:101083.10.1016/j.nmni.2023.101083.

125. Simeneh E, Gezimu T, Woldemariam M, Alelign D. Magnitude of Multidrug-resistant Bacterial Uropathogens and Associated Factors in Urinary Tract Infection Suspected Adult HIV-Positive Patients in Southern Ethiopia. *The Open Microbiology Journal*. 2022;**16**(1).10.2174/18742858-v16-e2208180.

126. Seid M, Markos M, Aklilu A, Manilal A, Zakir A, Kebede T, et al. Community-Acquired Urinary Tract Infection Among Sexually Active Women: Risk Factors, Bacterial Profile and Their Antimicrobial Susceptibility Patterns, Arba Minch, Southern Ethiopia. *Infect Drug Resist*. 2023;**16**:2297-310.10.2147/IDR.S407092.

127. Teferi S, Sahlemariam Z, Mekonnen M, Tamrat R, Bekana T, Adisu Y, et al. Uropathogenic bacterial profile and antibiotic susceptibility pattern of isolates among gynecological cases admitted to Jimma Medical Center, South West Ethiopia. *Sci Rep*. 2023;**13**(1):7078.10.1038/s41598-023-34048-4.

128. Mitiku A, Aklilu A, Tsalla T, Woldemariam M, Manilal A, Biru M. Magnitude and antimicrobial susceptibility profiles of Gram-Negative bacterial isolates among patients suspected of urinary tract infections in Arba Minch General Hospital, southern Ethiopia. *PLoS One*. 2022;**17**(12):e0279887.10.1371/journal.pone.0279887.

129. Beshah D, Desta AF, Woldemichael GB, Belachew EB, Derese SG, Zelelie TZ, et al. High burden of ESBL and carbapenemase-producing gram-negative bacteria in bloodstream infection patients at a tertiary care hospital in Addis Ababa, Ethiopia. *PLoS One*. 2023;**18**(6):e0287453.10.1371/journal.pone.0287453.

130. Beyene D, Bitew A, Fantew S, Mihret A, Evans M. Multidrug-resistant profile and prevalence of extended spectrum β-lactamase and carbapenemase production in fermentative Gram-negative bacilli recovered from patients and specimens referred to National Reference Laboratory, Addis Ababa, Ethiopia. *PLoS One*. 2019;**14**(9):e0222911.10.1371/journal.pone.0222911.

131. Bitew A, Adane A, Abdeta A. Bacteriological spectrum, extended-spectrum β-lactamase production and antimicrobial resistance pattern among patients with bloodstream infection in Addis Ababa. *Sci Rep*. 2023;**13**(1):2071.10.1038/s41598-023-29337-x.

132. Endaylalu K, Abera B, Mulu W. Extended spectrum beta lactamase producing bacteria among outpatients with ear infection at FelegeHiwot Referral Hospital, North West Ethiopia. *PLoS One*. 2020;**15**(9):e0238891.10.1371/journal.pone.0238891.

133. Zeynudin A, Pritsch M, Schubert S, Messerer M, Liegl G, Hoelscher M, et al. Prevalence and antibiotic susceptibility pattern of CTX-M type extended-spectrum β-lactamases among clinical isolates of gram-negative bacilli in Jimma, Ethiopia. 2018;**18**:1-10

134. Mulisa G, Selassie L, Jarso G, Shiferew T, Zewdu A, Abebe W, et al. Prevalence of extended Spectrum Beta-lactamase producing Enterobacteriaceae: a cross sectional study at Adama hospital, Adama, Ethiopia. 2016;**1**(1):1-6

135. Moges F, Eshetie S, Abebe W, Mekonnen F, Dagnew M, Endale A, et al. High prevalence of extended-spectrum beta-lactamase-producing Gram-negative pathogens from patients attending Felege Hiwot Comprehensive Specialized Hospital, Bahir Dar, Amhara region. 2019;**14**(4):e0215177
