# Supplementary material for: Colonization with extended-spectrum β-lactamase and carbapenemase-producing Enterobacterales in Ethiopia: A systematic review and meta-analysis
Source: PLoS One. 2025 Apr 1;20(4):e0316492. doi: 10.1371/journal.pone.0316492 (PMC11960885; doi:10.1371/journal.pone.0316492)
Supplement: S4 Figs — (DOCX) [file pone.0316492.s004.docx]

Supplementary file 4: the colonization rate of ESBL-and carbapenemase producing bacteria

o


Other= Enterobacter spp. (31), Citrobacter spp. (30) and Proteus spp.(11)

Other= Enterobacter spp. (6), Citrobacter spp. (5) and Proteus spp.(3)
