# Supplementary material for: Colonization with extended-spectrum β-lactamase and carbapenemase-producing Enterobacterales in Ethiopia: A systematic review and meta-analysis
Source: PLoS One. 2025 Apr 1;20(4):e0316492. doi: 10.1371/journal.pone.0316492 (PMC11960885; doi:10.1371/journal.pone.0316492)
Supplement: Attachment — Submitted filename: Response to Reviewers.docx [file pone.0316492.s005.docx]

**Response to Reviewers**

**Authors: We appreciate for spending your precious time and forwarding your valuable comments, which have significantly improved our manuscript. We are also grateful for this positive feedback. Please see below, bold, for a point-by-point response to the reviewers. We've copied your comments and responses below to make things easier for you. All line numbers refer to the revised manuscript file.**

Reviewer's Responses to Questions

**Comments to the Author**

1. Is the manuscript technically sound, and do the data support the conclusions?

The manuscript must describe a technically sound piece of scientific research with data that supports the conclusions. Experiments must have been conducted rigorously, with appropriate controls, replication, and sample sizes. The conclusions must be drawn appropriately based on the data presented.

Reviewer #1: Partly

Reviewer #2: Yes

Reviewer #3: Partly

2. Has the statistical analysis been performed appropriately and rigorously?

Reviewer #1: I Don't Know

Reviewer #2: Yes

Reviewer #3: No

3. Have the authors made all data underlying the findings in their manuscript fully available?

The [PLOS Data policy](http://track.editorialmanager.com/CL0/http:%2F%2Fwww.plosone.org%2Fstatic%2Fpolicies.action%23sharing/1/010f01940928c44a-ce96e1b6-1ebf-401b-8998-390dcaf30111-000000/DVWhCWYEXoU6-pDxHc4vX18bHFg6AqtzY4tMF2ejeiM=190) requires authors to make all data underlying the findings described in their manuscript fully available without restriction, with rare exception (please refer to the Data Availability Statement in the manuscript PDF file). The data should be provided as part of the manuscript or its supporting information, or deposited to a public repository. For example, in addition to summary statistics, the data points behind means, medians and variance measures should be available. If there are restrictions on publicly sharing data—e.g. participant privacy or use of data from a third party—those must be specified.

Reviewer #1: Yes

Reviewer #2: Yes

Reviewer #3: Yes

4. Is the manuscript presented in an intelligible fashion and written in standard English?

PLOS ONE does not copyedit accepted manuscripts, so the language in submitted articles must be clear, correct, and unambiguous. Any typographical or grammatical errors should be corrected at revision, so please note any specific errors here.

Reviewer #1: No

Reviewer #2: Yes

Reviewer #3: Yes

5. Review Comments to the Author

Please use the space provided to explain your answers to the questions above. You may also include additional comments for the author, including concerns about dual publication, research ethics, or publication ethics. (Please upload your review as an attachment if it exceeds 20,000 characters)

**Authors: Thank you for your positive feedback; we appreciate your feedback. We have revised the entire manuscript as necessary and have attempted to address the comments from the reviewers.**

**Reviewer #1:** This is a systematic review and meta-analysis of colonisation rates of extended-spectrum beta-lactamase-producing and carbapenemase-producing enterobacterales in Ethiopia. These are bacterial infections that are resistant to common antibiotics and can spread in healthcare and non-healthcare settings. They are usually harmless to healthy colonised individuals, but they can be dangerous to vulnerable patients. The review seems to be conducted and reported well, but the writing could be improved in places and should be checked throughout. I have a few comments.

**Authors: Thank you for your positive feedback.**

**Reviewer #1:** Abstract:

The conclusion in the abstract does not clearly represent the results of the meta-analysis, as it does not summarise the conclusion in the main paper. A summary could include something along the lines of “around a quarter of Ethiopians are colonised with ESBL-PE, and around one in 25 is colonised with CPE.” It should also make clear that it is based on a meta-analysis of studies judged to be of moderate-to-low risk of bias, and that the ESBL-PE study results were highly varied (high heterogeneity).

**Authors: Thank you for your input. We have accepted your comments. We have revised the conclusion in both the abstract and the main document to ensure clarity. (Please refer to the revised manuscript's conclusion section in the abstract).**

**Reviewer #1:** Introduction:
Line 82 – Should this be ESBL-PE rather than ESBLs?
Line 90 – It might be good to add that this is “in various specimens” here and add “using faecal specimens or swabs” to line 102.

**Authors: Thank you for your positive feedback. We have rewritten these sentence as you modified (Please refer to the revised manuscript line # 85, 98-99 &105).**

**Reviewer #1:** **Lines 100 to 116 – It would be good to explain, somewhere, why this has changed from the protocol, which specifies hospitalised patients in Africa.**

**Authors: Thank you for reflecting on your concern. A systematic review was conducted on hospitalized patients worldwide, with results stratified by continent. Consequently, we have revised the protocol to focus on all patients in Ethiopia, removing restrictions on the study setting.**

**Reviewer #1**: Methods:

Line 116 – see SF1 for a completed checklist.

**Authors: Thank you for bringing this issue to our intention. We have checked and updated it to the 2020 PRISMA completed checklist (Please refer to the supplementary file 1 of revised manuscript).**

**Reviewer #1:** Line 118 - Are these examples (electronic databases such as) or the full list of databases? All the sources searched should be clearly reported here or in the supplement.

**Authors: Thank you for your positive feedback. We listed these electronic databases as examples because we attempted to search other electronic databases. However, now we are considering PubMed, Hinari, and Google Scholar as the complete list of databases since all 527 records were identified from these three sources (Please refer to the revised manuscript line # 112-113 & Fig 1).**

**Reviewer #1:** Line 136 – the “No” doesn’t make sense here – should it be “Non-laboratory-based studies that reported…”?
**Authors: Thank you for your input. We corrected a typing error, changing "**No**" to "All" because; we used laboratory-based studies. Please refer to the revised manuscript line # 131).**

**Reviewer #1:** Line 156 – Was each record screened twice – once by each reviewer? How were differing decisions resolved? Or was each record screened by one reviewer?

**Authors: Thank you for your positive feedback. The data were screened independently twice by two reviewers (MT and MA). Discrepancies were resolved through discussion, and unresolved differences were resolved by consulting a third reviewer (GG) (Please refer to the revised manuscript line # 148-150).**

**Reviewer #1:** Line 170 – This sentence doesn’t make sense should it be …tools had 9 and 11 questions…?

**Authors: Thank you for your input; we appreciate your feedback. We have accepted your comment and we tried to amend it. (Please refer to the revised manuscript line # 164-165).**

**Reviewer #1:** Lines 171 to 172 – This sentence is not needed as this is explained in the next sentence.

**Authors: Thank you for raising this interesting point. We have accepted your comment and removed this sentence (Please refer to the revised manuscript line # 165-166).**

**Reviewer #1:** Lines 186 to 188 – It would be good to clearly report the changes to the protocol. The plan was for subgroups by region, strain and year of publication, so this adds setting, age and methods of outcome confirmation and removes strain and year of publication.
**Authors: Thank you for reflecting on your concern. We included a subgroup analysis by year of publication; however, subgroup analysis by strain is less important, as it may be cumbersome and the pooled prevalence for each strain has already been performed (Please refer to the revised manuscript table 7 and supplementary file 3).**

**Reviewer #1:** Results:
Lines 201 to 205 – There seems to be an error in the numbers reported here as 135 minus 96, 24 and 3 is 12 not 15. In the flow chart the 96 is 93.

**Authors: Thank you for your input; we appreciate your feedback. We corrected a typing error; changing "**96**" to "93"(Please refer to the revised manuscript line # 189).**

**Reviewer #1:** Discussion:
Line 361 – This sentence needs to be rewritten: CDT (31.2%) was comparable, and the lowest pooled estimate was…

**Authors: Thank you for your input. We have re-written and modified the whole paragraph (Please refer to the revised manuscript line # 352-357).**

**Reviewer #1:** Lines 378 to 383 - The conclusion in the paper is appropriate, but it could be written more clearly. It should also make clear that it is based on studies judged to be of moderate-to-low risk of bias, and that the ESBL-PE estimate has high heterogeneity.

**Authors: Thank you for bringing this issue to our intention. We have revised the conclusion in both the abstract and the main document to ensure clarity (Please refer to the revised manuscript's conclusion section in the abstract and the main document).**

**Reviewer #1:** Figure 1. This indicates that all the 527 records were identified from PubMed, Google Scholar and Hinari – were there none from the other databases or were they not searched?

**Authors: Thank you for your positive feedback. We attempted to search other databases but did not incorporate them into our text and figures because our focus was on PubMed, Google Scholar, and Hinari, as these three major databases were sufficient. Therefore, all 527 records were identified from these three databases.**

**Reviewer #1:** General:
Just a question, as I’m not a statistics expert, but is the probability reported the p-value for the heterogeneity or for the effect? For example, in Figure 3, the I-squared is zero, so no heterogeneity, the p-value is not significant (which is true for no significant heterogeneity), but the diamond doesn’t cross zero (the line of no effect) so should the p-value for the effect be significant?

**Authors: Thank you for your comments and questions. I² is zero, indicating no heterogeneity among the studies. The p-value for heterogeneity is not significant, supporting no significant heterogeneity. The diamond does not cross zero, suggesting a statistically significant effect (the confidence interval does not include the line of no effect). If the diamond does not cross zero, it means the confidence interval does not include the null effect (typically "no effect" is represented by zero for mean differences or one for ratios). This implies that the result is statistically significant. Generally, the lack of heterogeneity (I² = 0 and non-significant heterogeneity p-value) strengthens the conclusion, as it suggests consistency across studies and no distortion of the pooled effect.**

**Reviewer #1:** Were there any studies that did not meet the quality criterion? It seems that all fifteen studies were at moderate or low risk of bias, so I assume that no studies were actually excluded due to a high risk of bias? If this is the case, it might be good to state it clearly at the beginning of the results section, with a summary of the risk of bias results. I hope that my comments are helpful.

**Authors: Thank you for raising this interesting point. We incorporated the risk of bias results at the beginning of the results section. All fifteen studies were at moderate or low risk of bias; therefore, no studies were excluded due to a high risk of bias (Please refer to the revised manuscript line # 196-199).**

**Comments raised by Reviewer 2**

**Reviewer #2:** 1: A few places, the wording is unclear:
Line 136-139 – No laboratory-based studies have reported the presence of extended-spectrum beta-lactamase-producing and/or carbapenemase-producing Enterobacterales in human fecal specimens or rectal/perirectal swabs without restriction on publication year, and studies have been conducted in Ethiopia.

**Authors: Thank you for your positive feedback. We corrected a typing error, changing "**No**" to "All" because; we used laboratory-based studies (Please refer to the revised manuscript line # 131).**

**Reviewer #2:** Line 170 – The quality assessment tools were 9 and 11 for prevalence and cohort studies, respectively.

**Authors: Thank you for your input. We have accepted your comment and we tried to amend it. (Please refer to the revised manuscript line # 164-165).**

**Reviewer #2:** Line 378-383– Conclusion and recommendations section

**Authors: Thank you for bringing this issue to our intention. We have revised the conclusion in both the abstract and the main document to ensure clarity (Please refer to the revised manuscript's conclusion section in the abstract and the main document).**

**Reviewer #2:**  Line 202 and flow chart: There is a mismatch between the flow chart and the text. When doing the calculations, the flowchart match perfectly, by subtracting 93 (43 + 36 + 14) from 135, with 42 studies left. In the text, however, 93 has been replaced by 96. 96 should probably be 93, then everything fits perfectly.

**Authors: Thank you for your input; we appreciate your feedback. We corrected a typing error; changing "**96**" to "93"(Please refer to the revised manuscript line # 189).**

**Comments raised by Reviewer 3**

**Reviewer #3:** The systematic review appeared to be fairly comprehensive with search strategy, eligibility checks, main outcomes checked of the colonization rate of ESBL-PE and CPE from human fecal/rectal swab specimens in Ethiopia, data extraction and management outlines according to PRISMA and quality of study assessment using standard critical appraisal tools prepared by the Joanna Briggs Institute (JBI) for prevalence and cohort studies as is the case in this application. The data analysis endpoint was mainly colonization rate of ESBL-PEs and CPEs determined separately via a random effects model which is certainly appropriate in this context. Heterogeneity was assessed via the I square statistic. Sensitivity analysis was conducted to determine the influence of single studies on the pooled estimates. Publication bias and small study effects were checked by using a funnel plot test and Egger’s test. It appears that all the elements of this systematic review and meta-analysis were in place. The manuscript was well organized.

**Authors: Thank you for your positive feedback.**

**Reviewer #3:** When evaluating the publication bias the authors note that the ESBL-PE analysis revealed an asymmetric funnel plot, which indicates the presence of publication bias (Fig. 4). Similarly, Egger’s test revealed significant publication bias (P=0.004), as indicated in Table 3. Also in Fig 5. the Funnel plot showed publication biases related to the colonization of CPE. The investigators should explain more completely, if possible, the sources of the asymmetry in both plots.

**Authors: Thank you for bringing this issue to our intention. We have performed a trim-and-fill analysis to address the observed publication bias (Please refer to the revised manuscript line # 264-269, table 4 and 5).**

**Reviewer #3:** Also the interpretation of sensitivity is not detailed sufficiently for the reader in Table 4. In addition, in Table 5, the possible sources of heterogeneity, especially for those with an I squared above 90 and statistically significant (p<0.05) should be explained, if possible, for the reader.

**Authors: Thank you for reflecting on your concern. We have sought to interpret the sensitivity analysis with sufficient detail (Please refer to the introduction portion of revised manuscript line # 275-278).**
**Reviewer #3:** The investigators use the statistic, ES, on the Forest plots throughout the manuscript and in supplemental file 3. Explain that it is the colonization rate. If otherwise, please define it. Also the manuscript should be edited to be sure the reader knows what the p-values are measuring, much like the authors did for explaining the p-values associated with heterogeneity and publication bias.

**Authors: Authors: We appreciate your feedback. We have added colonization rate (Please refer to the revised manuscript)**

**Authors**: **Finally, we would like to say thank you for reviewing our work and making insightful suggestions and comments that helped to strengthen our manuscript. We have revised the manuscript as necessary.**
