# Supplementary material for: Colonization with extended-spectrum β-lactamase and carbapenemase-producing Enterobacterales in Ethiopia: A systematic review and meta-analysis
Source: PLoS One. 2025 Apr 1;20(4):e0316492. doi: 10.1371/journal.pone.0316492 (PMC11960885; doi:10.1371/journal.pone.0316492)
Supplement: Attachment — Submitted filename: Response_to_Reviewers_auresp_2.docx [file pone.0316492.s006.docx]

**Response to Academic editor and Reviewers**

**Authors: We appreciate for spending your precious time and forwarding your valuable comments, which have significantly improved our manuscript. We are also grateful for this positive feedback. Please see below, bold, for a point-by-point response to the academic editor and reviewers. We've copied your comments and responses below to make things easier for you. All line numbers refer to the revised manuscript file.**

**Academic editor:**

**Journal Requirements:** Please review your reference list to ensure that it is complete and correct. If you have cited papers that have been retracted, please include the rationale for doing so in the manuscript text, or remove these references and replace them with relevant current references. Any changes to the reference list should be mentioned in the rebuttal letter that accompanies your revised manuscript. If you need to cite a retracted article, indicate the article’s retracted status in the References list and also include a citation and full reference for the retraction notice.

**Authors: Thank you for your positive feedback. We have amended the reference list according to PLOS ONE’s criteria, and we ensure that it is complete and correct.**

**Reviewer #1:** Thank you for addressing my comments. There are two queries that could be clarified further (points 1 and 2 below), and five minor points (3 to 7 below).

**Authors: Thank you for your input. We have accepted your comments and revised the entire document accordingly. We appreciate the opportunity to learn from your feedback.**

**Reviewer #1:** Thank you for addressing my comments. There are two queries that could be clarified further (points 1 and 2 below), and five minor points (3 to 7 below).
1. The p-values that are reported in brackets after the effect estimates appear to be probabilities for the I-squared heterogeneity measure. For example, 4.4% (95% CI: 0.9–7.9%, P = 0.636) – the effect estimate probability is 0.01, while the I-squared probability is 0.64.
ChatGPT provided this example to explain how the probability for I-squared should be distinguished from the probability for the effect estimate:
The pooled odds ratio (OR) for the intervention’s effect on mortality was 0.78 (95% CI: 0.65–0.93, p = 0.004), indicating a significant reduction in risk. However, substantial heterogeneity was observed (I² = 67%, p < 0.01), suggesting variation among studies.
If you use the p for heterogeneity in brackets after the effect estimate, at a minimum, it requires I² before it, otherwise it appears to be the p for the effect estimate.

**Authors: The p-values reported in brackets after the effect estimates represent the probabilities for the I-squared heterogeneity measure. To clarify, we have added "I²" before all p-values. Please refer to the revised manuscript's abstract and results sections.**

**Reviewer #1:** 2. As required by item 24c in the PRISMA checklist, any changes to those methods that were prespecified in the protocol, should be reported in the paper or a supplementary file.

**Authors: Thank you for your input. We have accepted your comments and revised the Methods section to report the deviations from the original protocol. Please refer to the revised manuscript (lines 111–125).**

**Reviewer #1:** 3. Line #131 has been revised, but it is not a sentence. It should start with “We included…” or should contain a statement that these studies were included.

**Authors: Thank you for your positive feedback. We have rewritten these sentence as you modified (Please refer to the revised manuscript line # 146).**

**Reviewer #1:** **4. In Table 1, what does the “no of cases” refer to? How does this differ from the Total ESBL-PE? How can the number of cases be fewer than the total ESBL-PE (see the first line, Temsegen)?**

**Authors: Thank you for reflecting on your concern. We attempt to clarify this issue: the number of cases refers to the number of participants. The number of cases may be lower than the number of ESBL-PE and CPE because some study participants may be colonized by two or more ESBL-PE and CPE.**

**Reviewer #1**: 5. Lines 303 and 358 – according to the figure, the 55.22 should be 55.25.

**Authors: Thank you for bringing this issue to our intention. We have checked and corrected it to the 55.22 to 55.25 (Please refer to the revised manuscript line # 52, 319 & 374).**

**Reviewer #1:** 6. Line 345 - when comparing EBSL-PE and CPE in brackets it should be (29.9% and 4.6%) rather than vs to match the text where the comparison is between hospital versus community.

**Authors: Thank you for your positive feedback. We corrected this language error (Please refer to the revised manuscript line # 361).**

**Reviewer #1:** 7. Line 351 - I think this should be ESBL-PE rather than just ESBL.

**Authors: Thank you for your input. We corrected a typing error, changing "ESBL" to "ESBL-PE" (Please refer to the revised manuscript line # 367).**

**Authors**: **Finally, we would like to say thank you for reviewing our work and making insightful suggestions and comments that helped to strengthen our manuscript. We have revised the manuscript as necessary.**
